# Real Clinical Practice and Therapeutic Management Following COVID-19 Crisis in two Hospitals in Iran: A Statistical and Conceptual View

**Published:** 2020-11

**Authors:** Ali Najafi, Mostafa Ghanei, Ghasem Janbabaei, Ali Akbar Velayati, Seyed Hassan Saadat, Hamidreza Jamaati, Payam Tabarsi, Farzaneh Dastan, Malihe Ram, Enayat Darabi, Saeid Fathi, Mohammad Gholami Fesharaki, Amir Hosein Ghazale, Shahrzad Saloo

**Affiliations:** 1 Molecular Biology Research Center, Systems Biology and Poisonings Institute, Baqiyatallah University of Medical Sciences, Tehran, Iran,; 2 Chemical Injuries Research Center, Systems Biology and Poisonings Institute, Baqiyatallah University of Medical Sciences, Tehran, Iran,; 3 Department of Hematology Oncology, Tehran University of Medical Sciences, Tehran, Iran,; 4 Clinical Tuberculosis and Epidemiology Research Center, National Research Institute of Tuberculosis and Lung Diseases (NRITLD), Shahid Beheshti University of Medical Sciences, Tehran, Iran,; 5 Behavioral Sciences Research Center, Lifestyle Institute, Baqiyatallah University of Medical Sciences, Tehran, Iran,; 6 Chronic Respiratory Diseases Research Center, NRITLD, Shahid Beheshti University of Medical Sciences, Tehran, Iran,; 7 Department of Biostatistics, Ferdows Paramedical School, Birjand University of Medical Sciences, Birjand, Iran,; 8 School of Public Health, Tehran University of Medical Sciences, Tehran, Iran,; 9 Postdoctoral Fellow, University of Tehran, Tehran, Iran,; 10 Biostatistics Department, Faculty of Medical Sciences, Tarbiat Modares University, Tehran, Iran,; 11 Student Research Committee, Baqiyatallah University of Medical Sciences, Tehran, Iran.

**Keywords:** COVID-19, Treatment Strategies, Hospital Information System (HIS)

## Abstract

**Background:**

The Coronavirus disease 2019 (COVID-19) outbreak quickly has spread and became a pandemic. However, no approved therapeutics or effective treatment is available for the treatment of these patients. The present study was done to retrospectively assess the treatment strategies (e.g., pharmaceutical care services) for COVID-19 patients in selected hospitals and highlight the importance of such services in the management of a pandemic.

**Materials and Methods:**

Data from a series of COVID-19 patients (978 patients; 658 males [66.9%] and 324 females [33.1%]) admitted to the selected hospitals in Tehran from 20 February to 19 March 2020 were retrieved retrospectively from the Health Information System (HIS) of the hospitals. The statistical tests were used for analyzing the effect and correlation of the variables (drugs) with the average length of stay (ALOS) in the hospital.

**Results:**

Diverse medication classes and old drugs with or without strong evidence of therapeutic effects against the novel coronavirus, some previously tried as a treatment for SARS-CoV and MERS-CoV, were mostly used for the treatment of patients in the hospitals. Many medications (broad-spectrum antibiotics and antivirals) or combination therapies are used without evidence of their therapeutic effects during pandemics.

**Conclusion:**

Therefore, guidelines should be provided for the off-label use of these drugs by policymakers and stakeholders during a pandemic emergency due to high demands. Also, monitoring of the HIS data can play an important role in improving public health response to emerging diseases.

## INTRODUCTION

The coronavirus disease 2019 (COVID-19) outbreak has quickly spread and became a pandemic. It is considered as an important global public health emergency. Nonetheless, no approved treatments are available with clinically considerable efficacy for the treatment of patients suffering from the disease. As a result, the health centers of different countries have promulgated different treatment guidelines and therapeutic strategies for managing viral disease. Different pharmacological agents have been recommended for the treatment of COVID-19, including antiviral agents (e.g., lopinavir/ritonavir, remdesivir, ribavirin, and arbidol, etc.), antibiotics, anti-inflammatory drugs, antimalarial drugs (chloroquine and hydroxychloroquine), intravenous immunoglobulin (IVIG), and angiotensin receptor blockers (losartan), etc. (1–4). Pharmaceutical care services could play a vital role in the treatment of COVID-19 patients through the use of patient-focused pharmaceutical care frameworks, including evidence-based decisions regarding cumulative experience, monitoring of drug safety and efficacy for patients (e.g., critically ill patients, combined underlying diseases, etc.) and the special population (e.g., pregnant cases, those with impaired immunity, elderly patients, children, etc.), and management of drug interactions. This can improve the treatment outcomes of COVID-19 patients. However, it is uncertain that to what extent these measures can lead to patient recovery.

The present study was conducted to retrospectively assess the treatment strategies (e.g., pharmaceutical care services) for COVID-19 patients in selected hospitals and to highlight the importance of pharmaceutical care services in the management of a pandemic. This study also highlights the role of Hospital Information System (HIS) in improving public health response to emerging diseases.

## MATERIALS AND METHODS

Data from a series of COVID-19 patients (978 patients; 658 males [66.9%] and 324 females [33.1%]) with a mean age of 55.55±15.60 years admitted to the selected Hospitals (Masih Daneshvari and Baqiyatallah hospitals) in Tehran from 20 February to 19 March 2020 were retrieved retrospectively from the HIS of the hospitals. Based on the observational data obtained from the HIS of these hospitals, most of the patients lived in Tehran (n=833, 85.2%). The Iranian Ministry of Health and Medical Education (MOHME) centralized COVID-19 patients’ treatment in some selected hospitals, including Masih Daneshvari Hospital, which is a tuberculosis and respiratory diseases referral center in the country. This hospital followed a broad-spectrum antimicrobial regimen for the treatment of COVID-19 patients. Diverse medication classes and old drugs with or without strong evidence of therapeutic effects against the novel coronavirus, some previously tried as a treatment for SARS-CoV and MERS-CoV, were mostly used for the treatment of patients in the hospital. These medications include antivirals, antibiotics, anti-inflammatory drugs, antimalarial drugs, intravenous immunoglobulin (IVIG), and other supportive treatment regimens. In addition to these treatment strategies, the most severely ill patients received supplemental oxygen and ventilation support.

Pearson's correlation coefficient (r) and Spearman’s rank correlation test were used for analyzing the correlation of the variables (drugs) with the average length of stay (ALOS) in the hospital. Furthermore, the t-test and ANOVA were used to analyze the correlation of gender with the ALOS.

## RESULTS

### 1. Pharmaceutical care for hospitalized patients with COVID-19 in studied hospitals

#### 1.1. The available evidence and association of the treatment strategies with the ALOS

Despite the possible existence of the disease in the country before 19th February, Iran reported its first 2 cases of COVID-19 on February 19, 2020, in Qom. On February 20, there was already a huge surge in the number of hospitalized patients in Tehran, especially in Masih Daneshvari hospital, probably due to lack of social distancing, etc.

**Figure 1.**
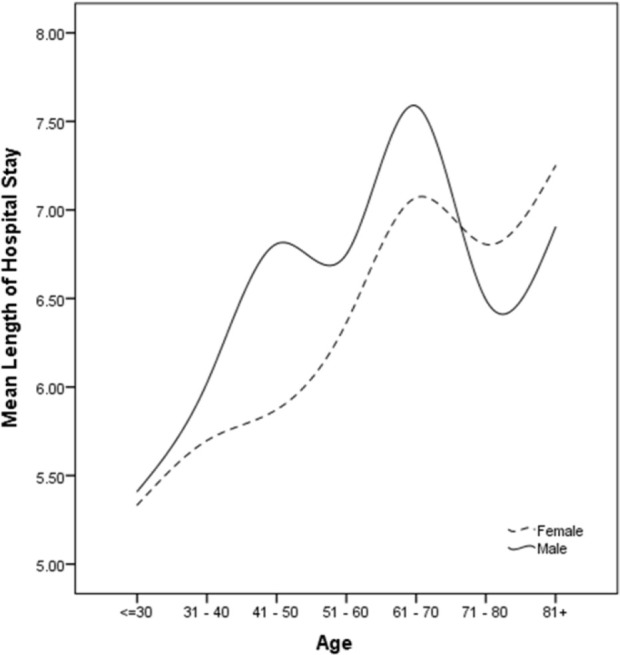
Average length of stay in hospital based on the age

The number of hospitalizations increased exponentially. The age of the hospitalized patients ranged from 50 to 64 years, and there was approximately a twofold increase in the hospitalization rate in males compared with females. The data retrieved from the HIS of the hospital indicated that the increase in the ALOS was associated with an increase in age, but a small proportion of the young population, which accounts for about 50% of the country's total population, needed hospitalization. A larger proportion of hospitalized patients consisted of the middle-aged population, which makes up about 50% of the country's remaining population (Figure 1). However, a sharp decrease in the rate of hospitalization was observed in the age group 65 to 85 years. The hypothetically lower rate of hospitalization in children and young adults may be associated with their vaccination status against other diseases. Furthermore, a lower hospitalization rate among older patients (65–85 years) may suggest anergy induction (immune unresponsiveness), needing further investigation.

**Figure 2.**
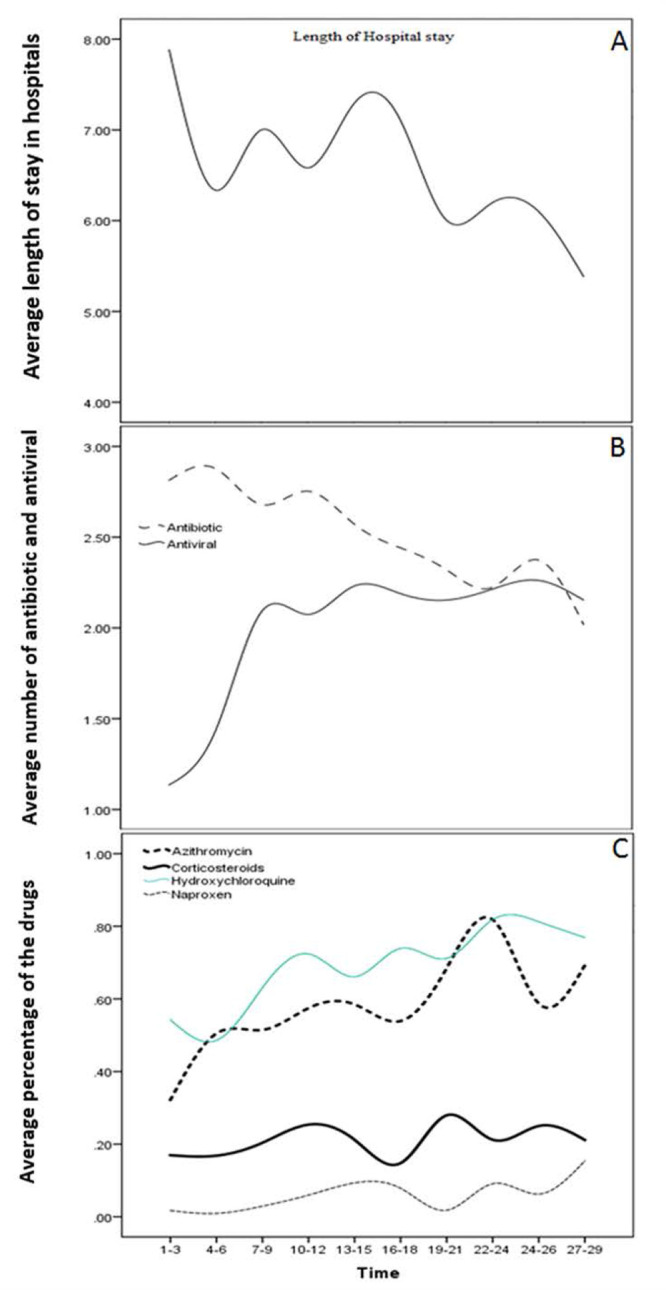
A: the downward trend in ALOS from 20 February to 19 March 202.; B: Average number of antibiotic and antiviral used in the hospital (B); C: Average percentage of the drugs used in hospital

A declining trend was observed in the ALOS from 20th February to 19th March 2020, probably due to the management experiences gathered from the previous days, the nature of the virus, national interim clinical guidance, and the World Health Organization recommended interventions. The mean ALOS was 6.62±3.85 during this period. Figure 2A shows the downward trend in the ALOS over a month. The mean ALOS for patients over 40 years of age was one day longer than the patients under 40 years of age (6.13±3.79 and 6.89±3.87, respectively; P=0.003).

With increased experience in the management of the disease, the medical team was able to better characterize the COVID-19 patients and make a timely diagnosis of the disease, and the therapeutic strategies also improved one month after the outbreak. Also, during this time, Iran launched a series of emergency and supportive therapies for the management of patients suffering from COVID-19. The aforementioned factors led to a decrease in ALOS in late March; however, the number of hospitalized patients increased from 20th February to 19th March 2020 (Figure 2 A). Nonetheless, it should be noted that the available guidelines did not provide detailed suggestions on the therapeutic strategies. In this case, patient-centered interdisciplinary collaboration and management are of great benefit to both the patients and the society and can help improve the outcome and decrease the mortality rate in COVID-19 patients.

The frequency and concomitant use of drugs among the patients are represented in Tables 1 and 2. Among the hospitalized patients, 958 cases (97.96%) received antibiotics, 908 cases received antivirals (92.85%), 674 patients (68.92%) received antimalarial drugs, 208 patients (21.26) received corticosteroid, and 72 cases received IVIG (7%).

**Table 1.** Frequency of medication used among patients.

**Drug**	**N**	**%**	**Drug**	**N**	**%**
**Oseltamivi Phosphate**	898	92%	**Epinephrine**	82	8%
**Lopinavir Ritonavir**	820	84%	**Hydrocortisone**	79	8%
**Pantoprazole**	733	75%	**Dexamethasone**	75	8%
**Acetaminophen**	720	74%	**Intravenous immunoglobulin (IVIG)**	72	7%
**Antimalaria**	674	69%	**Midazolam**	71	7%
**Hydroxychloroquine**	674	69%	**Metoprolol**	71	7%
**Ceftriaxone**	585	60%	**Amlodipine**	68	7%
**Azithromycin**	574	59%	**Ipratropium Bromide/Salbutamol**	67	7%
**Enoxaparin**	518	53%	**Diphenhydramine**	66	7%
**Meropenem**	461	47%	**Magnesium Sulfate**	65	7%
**Vancomycin**	440	45%	**Vitamin A&D**	65	7%
**Heparin**	404	41%	**Promethazine**	61	6%
**Levofloxacin**	303	31%	**Vitamin_B1**	59	6%
**Ribavirin**	258	26%	**Naproxen**	57	6%
**Losartan**	163	17%	**Nitrocontin**	57	6%
**Potassium_Chloride**	158	16%	**Fluticasone/salmeterol**	57	6%
**Dextromethorphan**	157	16%	**Ipratropium**	52	5%
**Atorvastatin**	155	16%	**Atropine**	50	5%
**Ondansetron**	155	16%	**Lactulose**	50	5%
**Asa**	146	15%	**Indomethacin**	47	5%
**Vitamin D3**	135	14%	**Metformin**	46	5%
**Lidocaine**	129	13%	**Vitamin Multi**	46	5%
**Codeine/paracetamol**	103	11%	**Prednisolone**	43	4%
**Furosemide**	103	11%	**Linezolid**	43	4%
**Fentanyl**	90	9%	**Ceftazidime**	41	4%

**Table 2.** Concomitant use of the four class of drug used by patients

**Concomitant use of the drugs**	**Antibiotic**	**Antiviral**	**Corticosteroids**	**Antimalarial**
**0**	20(%2.04)	70(%7.16)	770(%78.73)	304(%31.08)
**1**	92(%9.41)	89(%9.1)	162(%16.56)	674(%68.92)
**2**	461(%47.14)	567(%57.98)	36(%3.68)	-
**3**	213(%21.78)	252(%25.77)	9(%0.92)	-
**4**	144(%14.72)		1(%0.1)	-
**5**	44(%4.5)	-	-	-
**6**	3(%0.31)	-	-	-
**7+**	1(%0.10)	-	-	-
**Mean**	2.02	0.69	0.27	2.53
**SD**	0.79	0.46	0.58	1.10

The correlations between the ALOS and antibiotic, antiviral, and corticosteroid therapies are presented in Tables 3–5. As shown in Table 2, except for azithromycin and ceftriaxone, other antibiotic medications did not show a significant correlation with the ALOS, in other words, no positive clinical significance was observed by correlation analysis.

**Table 3.** The correlation between antibiotic drugs and length of stay in hospital

**Variables**	**Length of Stay in Hospital(1)**	**Ceftriaxone(2)**	**Azithromycin(3)**	**Meropenem(4)**	**Vancomycin(5)**	**Levofloxacin(6)**	**Linezolid(7)**	**Ceftazidime(8)**	**Clindamycin (9)**	**Co Trimoxazole (10)**	**Metronidazole (11)**	**Tetracycline (12)**	**Cefepime (13)**
**(1)**	1												
**(2)**	−0.37^**^	1											
**(3)**	−0.26^**^	0.76^**^	1										
**(4)**	0.46^**^	−0.52^**^	−0.36^**^	1									
**(5)**	0.42^**^	−0.69^**^	−0.41^**^	0.83^**^	1								
**(6)**	0.36^**^	−0.85^**^	−0.68^**^	0.35^**^	0.56^**^	1							
**(7)**	0.27^**^	−0.42^**^	−0.49^**^	0.11^**^	0.05^*^	0.54^**^	1						
**(8)**	0.00	0.02	−0.19^**^	−0.08^**^	−0.16^**^	0.07^*^	0.31^**^	1					
**(9)**	−0.07^*^	−0.07^*^	0.01	0.21^**^	0.27^**^	0.09^**^	−0.14^**^	−0.19^**^	1				
**(10)**	0.06^*^	−0.08^**^	−0.15^**^	0.41^**^	0.34^**^	0.03	−0.29^**^	0.07^*^	0.06^*^	1			
**(11)**	−0.05^*^	0.020	0.09^**^	0.50^**^	0.27^**^	−0.05	−0.10^**^	−0.13^**^	0.30^**^	0.33^**^	1		
**(12)**	0.08^**^	−0.14^**^	−0.06^*^	−0.03	0.08^**^	0.18^**^	−0.09^**^	0.01	−0.11^**^	−0.12^**^	−0.12^**^	1	
**(13)**	0.10^**^	−0.17^**^	−0.24^**^	0.07^*^	0.16^**^	0.36^**^	0.30^**^	0.02	0.14^**^	0.16^**^	0.07^*^	−0.04	

* <0.5;

** <0.01

**Table 4.** The correlation between antiviral drugs and length of stay in hospital

	**Length of Stay in Hospital(1)**	**Oseltamivir Phosphate (2)**	**Lopinavir/ritonavir (3)**
**Length of Stay in Hospital(1)**	1		
**Oseltamivir Phosphate (2)**	−0.25^**^	1	
**Lopinavir/ritonavir (3)**	−0.19^**^	0.86^**^	1
**Ribavirin (4)**	0.17^**^	0.49^**^	0.52^**^

* <0.5;

** <0.01

**Table 5.** The correlation between corticosteroids drugs and length of stay in hospital

	**(1)**	**(2)**	**(3)**	**(4)**	**(5)**	**(6)**	**(7)**	**(8)**
**Length of Stay in Hospital(1)**	1							
**Dexamethasone (2)**	0.03	1						
**Hydrocortisone (3)**	0.00	−0.21^**^	1					
**Methylprednisolone (4)**	−0.19^**^	−0.19^**^	0.27^**^	1				
**Prednisolone (5)**	0.11^**^	0.18^**^	−0.12^**^	0.08^**^	1			
**Salmeterol Fluticasone (6)**	0.22^**^	−0.15^**^	−0.01	−0.12^**^	−0.09^**^	1		
**Budesonide (7)**	0.020	−0.12^**^	0.31^**^	−0.1^**^	0.06*	−0.03	1	
**Budesonide Formoterol (8)**	−0.050^*^	0.12^**^	−0.15^**^	−0.15^**^	−0.01	−0.11^**^	−0.1^**^	1

* <0.5;

** <0.01

Moreover, there was a significant correlation between azithromycin and ceftriaxone use and ALOS (r = 0.76, P <0.001; Table 3). Ceftriaxone was prescribed with azithromycin in most of the cases, which means that the reduction in the ALOS associated with ceftriaxone use may be due to the concomitant use of this drug with azithromycin.

Of the three antiviral drugs commonly prescribed (oseltamivir phosphate, lopinavir/ritonavir, and ribavirin), only oseltamivir and lopinavir/ritonavir relatively decreased ALOS (Table 4). Among the corticosteroid therapies, methylprednisolone had an increasing effect on the ALOS of patients with COVID-19 (Table 5). We observed a significant correlation between hydroxychloroquine use and ALOS (r=−0.15). This antimalarial drug was able to considerably reduce ALOS, which further presents evidence for its potential therapeutic effects against COVID-19. Furthermore, concomitant use of the hydroxychloroquine and azithromycin (411 cases; 42%) was associated with a shorter ALOS (r =−0.227; p = 0.001). Another commonly prescribed medicine was naproxen, but there was no correlation between naproxen use and ALOS during the study period.

In situations where standard therapies and guidelines are not available, hospital pharmacists can be of great importance in providing clinical evidence (i.e., patients' conditions) and guidance for formulating and adjusting therapeutic regimens. In the fight against the COVID-19 outbreak, hospitals are facing problems in terms of drug supply and pharmaceutical care and personnel.

## DISCUSSION

### 

#### 1.2. Antibiotics

A study conducted in Wuhan reported that 15% of hospitalized COVID-19 patients and half of the patients who died of the disease had secondary infections (5), indicating that appropriate antibiotic therapy may be useful in reducing mortality and morbidity associated with the disease. In the present study, in the studied hospital, azithromycin was used as adjunctive therapy for the treatment of COVID-19 patients (59% of patients) due to its immunomodulatory and anti-inflammatory effects. As shown in Tables 1 and 2, a wide range of antibiotics were used for the treatment of up to about 98% of the COVID-19 patients in our studied hospital. About 41% of the patients received 3 or more antibiotics due to pulmonary consolidation and the probable symptoms of viral pneumonia. However, there was a gradual decline in antibiotic use over time (Figure 2B). Based on the data obtained from the hospital, it is recommended that blind or inappropriate use of antibiotics should be avoided, particularly the broad-spectrum antibiotics, because their use may be associated with the inflammatory storm in the absence of bacterial infections via the direct stimulation of pro-inflammatory cytokines (e.g., IL-1β, IL-6, and TNF-α, etc.) and indirectly by inducing Toll-like receptor 4 expression and gut endotoxins production leading to endotoxemia (resulting in the upregulation of pro-inflammatory cytokines) (6–8). The COVID-19 pandemic, which has led to an increase in the use of broad-spectrum antibiotics (e.g., a popular combination therapy of hydroxychloroquine and azithromycin) may also lead to global antibiotic-resistance. Additionally, overuse may lead to a decreased effectiveness of the antibiotics (e.g., azithromycin). Therefore, the empirical use of antibiotics needs urgent de-escalation based on microbiological findings and clinical response.

It is noteworthy that there should be a rational use of drugs (e.g., antibiotics and antiviral, etc.) based on the individual’s clinical needs, with the lowest costs and by taking into consideration the harms related to the inappropriate use of medications in the COVID-19 pandemic.

#### 1.3. Antiviral agents

Antiviral and antimalarial drugs were widely recommended by the latest Iranian MOHME guidelines and doctors' opinions (Figure 2 B and C). In the studied hospital, the percentages of patients who received the antiviral and anti-malaria medications were as follows: oseltamivir (Tamiflu; 92%), lopinavir/ritonavir (84%), ribavirin (26%), and hydroxychloroquine (69%) (Table 1). The use of these medications was off-label. The concomitant use of three or more antiviral drugs is not recommended; however, about 33% of the patients received at least 3 or 4 types of antiviral drugs, which can cause adverse events in the COVID-19 patients. Based on the data presented, there was a sharp increase in the use of antiviral drugs during the first few weeks of the outbreak followed by a steady increase with a very gentle slope, indicating a reduction in the use of broad-spectrum antivirals over time (Figure 2B). It is noteworthy that drug stockpile, safety, and cost should be considered when prescribing antiviral drugs.

Despite the lack of strong evidence for its clinical outcomes, oseltamivir was widely prescribed in this hospital. Any empirical use of anti-influenza therapy requires microbiological and clinical evidence, but there were difficulties in distinguishing bacterial and viral cases of pneumonia and the novel coronavirus infection, as well as time-consuming laboratory diagnosis, leading to the increased use of the drug.

No significant antiviral effect of lopinavir/ritonavir (branded as Kaletra) has been reported among patients with severe COVID-19 (2), but there is evidence for its beneficial effect in secondary endpoints of a clinical trial among these patients (9). Likely, flawed methodology, such as sub-optimal methodological quality and inconclusive data can limit the level of confidence for justification; thus, further clinical confirmation of these medications is needed. It is also uncertain whether lopinavir/ritonavir monotherapy or its combination therapy with other antiviral drugs, as has been reported for SARS (10), can be beneficial for improving COVID-19 patients’ outcome. Although there was a correlation between the ALOS and oseltamivir and lopinavir/ritonavir in the present study, it should be taken into consideration that only correlation analysis for ALOS was performed, other outcomes, such as mortality rate were not available. Management of medication interactions is of great importance. The combination of lopinavir-ritonavir with other medications metabolized by the CYP3A enzyme (e.g., atorvastatin, midazolam, etc.) should be avoided.

Ribavirin, a guanosine analogue, was used in a smaller proportion of patients in the hospital. The potential adverse effects of this antiviral drug, including hematologic toxicity (hemolysis and anemia) outweigh its potential as an antiviral against COVID-19, thus limiting its clinical investigation (11). The combination of IFNα (IFNα2b) and IF Nβ1b with other antiviral agents (lopinavir/ritonavir, ribavirin and/or remdesivir), as has been reported for the treatment of COVID-19 (1) and in clinical trials (Open-label Randomized Controlled Trial: NCT04276688), with the aim of increasing the efficacy of the antiviral drugs remains to be further investigated. The safe use of drugs is of great importance and should be promoted through evidence-based investigations.

#### 1.4. Antimalarial drugs

Chloroquine and hydroxychloroquine have been used as anti-malarial drugs and for the treatment of autoimmune diseases. It has been suggested that these medications have a potential broad-spectrum antiviral effect (12–16). Hydroxychloroquine, a chloroquine analog, was widely used in the hospital due to the presence of evidence of its therapeutic effects (Figure 2 C) against SARS-CoV in vitro and COVID-19 infected Vero cells (3, 17).

Theoretically, the immunomodulatory effects of hydroxychloroquine and chloroquine may be capable of suppressing the immune response. The use of a combination regimen, including hydroxychloroquine and azithromycin (42% of patients), has been previously found to be associated with viral load reduction/disappearance in COVID-19 patients, but critical outcomes (e.g., death) were not indicated (18). Although the combination of hydroxychloroquine with other medications (42% of patients) was found to be associated with a decreased ALOS in the study hospital, current evidence does not completely support the level of confidence for appropriate justification of the beneficial use of quinine derivatives in patients suffering from COVID-19 due to the flawed methodologies (e.g., sub-optimal methodological quality) and inconclusive data in the literature. Therefore, further, high-quality studies and randomized controlled trials with **g**lobal cooperation are needed to provide clinical practice guidelines and information about other factors, such as mortality, morbidity, safety, etc. On the other hand, continuous telemetry (Mobile Cardiac Outpatient Telemetry) has been recommended for patients receiving this regimen due to a potential increase in cardiovascular risks (e.g. long QT syndrome [LQTS] and Torsades de pointes [TdP]),(19–21).

#### 1.5. Adjunctive therapies

##### 1.5.1 Corticosteroids

Corticosteroids were commonly used for the treatment of critically ill MERS patients (22). In the present study, corticosteroids were prescribed for 21.26% of patients in the studied hospital due to their anti-inflammatory effects and their ability to suppress cytokine storm in COVID-19 (dexamethasone [8%]; prednisolone [4%]; hydrocortisone [8%]; fluticasone/salmeterol [6%]; Table 1). Short-term use of corticosteroids (≤7 days) at low-to-moderate doses (e.g., ≤0.5–1 mg/kg per day methylprednisolone) may be considered for critically ill patients suffering from COVID-19; however, it is recommended that benefits and harm be weighed before use (benefit analysis). As advocated by some critical care specialists, corticosteroid therapy can be used for septic shock-reversal (refractory septic shock) and in patients with underlying diseases (asthma or chronic obstructive pulmonary disease). Also, it has been reported that corticosteroid therapy can decrease case-fatality risk (23, 24).

##### 1.5.2 IVIG

IVIGs have also been used as a potential adjunct therapy for various inflammation-related diseases and COVID-19 due to their anti-inflammatory effects and the passive immunization of patients. Due to their ability to suppress pathogenic cytokines and modulate the T-cell function and immunologic injury, IVIGs have been used for the treatment of hemophagocytic lymphohistiocytosis (HLH) and cytokine storm syndrome. IVIGs have also been associated with early recovery and shorter ALOS in COVID-19 patients (25–27). However, its use should be carefully considered due to its potential adverse effects, such as fever, renal failure, and myalgia (26). More clinical studies are needed to determine its clinical efficacy against COVID-19. Furthermore, the cost and availability of IVIGs may render them not suitable for use as a treatment strategy in emergency situations, such as the COVID-19 pandemic. In the present study, IVIGs were prescribed for 7% of patients due to the aforementioned difficulties, where its correlation analysis was not possible.

## CONCLUSION

One of the main limitations in this study was the lack of accessibility of the duration of antibiotic, antiviral, and antimalarial drug therapies, as well as the patients’ outcomes (e.g., mortality, laboratory findings, etc.) due to lack of data or missing data in the HIS of the hospital. From a broader perspective, efficient HIS can offer a unique opportunity by providing complete data integration for pharmacists to actively participate in the management and therapeutic approaches of patients. Pharmacists can help in supply chain management, and they can also help in resolving gaps in medications and drug safety. The participation of pharmacists will not only help to improve the patient’s outcome but also maximize their value as part of the healthcare team. On the other hand, efficient HIS can offer a unique opportunity for policymakers to obtain key surveillance data to provide better response to COVID-19 and future health emergencies.

Many medications (broad-spectrum antibiotics and antivirals) or combination therapies are being used without evidence of their therapeutic effects during pandemics. Therefore, guidelines should be provided for the off-label use of these drugs by policymakers and stakeholders during a pandemic emergency due to high demands. The emergency use of existing drugs outside global and national guidelines may be associated with side effects and waste of resources. The cost of some of these medications, such as the expensive antivirals, which may not be available for all patients, health inequities, and strain on the healthcare systems, can be linked to worsening outcomes during the COVID-19 pandemic. The day-to-day efforts of medical staff for the optimization of supportive treatment measures should not be underestimated.
